# Wet-spun Ag/PEDOT: PSS composite fibers for high-sensitive SERS sensing and high electrical conducting

**DOI:** 10.1038/s41598-024-80655-0

**Published:** 2024-11-25

**Authors:** Fan Wu, Haoyu Shi, Yulong Gao, Lin Cheng, Tongkai Gu, Tong Liu, Ziyun Chen, Wei Fan

**Affiliations:** 1 School of Textile Science and Engineering, Xi’an Polytechnic University, Xi’an, 710048 China; 2 Key Laboratory of Functional Textile Material and Product of Ministry of Education, Xi’an Polytechnic University, Xi’an, 710048 China; 3 State Key Laboratory of Dynamic Testing Technology, North University of China, Taiyuan, 030051 China; 4 School of Mechanical and Electrical Engineering, Xi’an University of Architecture and Technology, Xi’an, 710055 China; 5 State Key Laboratory for Manufacturing System Engineering, Xi’an Jiaotong University, Xi’an, 710054 China

**Keywords:** Flexible fiber, Wet spinning, PEDOT:PSS, Ag nanomaterials, Surface-enhanced Raman scattering, Materials for optics, Soft materials, Optical materials and structures, Optical materials, Soft materials

## Abstract

Nanometal-based composite fibers have been widely explored in flexible sensors due to their outstanding optical and electrical properties. However, the weak binding force between metallic nanomaterial and fiber greatly limits the real application. In this work, nano silver (Ag) are strongly bonded with poly(3,4-ethylenedioxythiophene)-poly (styrene sulfonate) (PEDOT: PSS) fiber by the wet-spun process. Ag-S chemical bonds are formed by the interaction of Ag and PEDOT. The Ag/PEDOT: PSS composite fiber shows excellent surface-enhanced Raman scattering (SERS) sensitivity on Rhodamine 6G (R6G) molecules. The detection limit can reach 10^–11^ M and Raman enhancement factor (EF) is of 1.3 × 10^7^. The high-sensitive SERS activity of Ag/PEDOT: PSS composite fiber mainly results from PEDOT: PSS, and the enhancement factor is 3 orders of magnitude better than that of other PEDOT: PSS based SERS substrates. Moreover, the composite fiber has metal-level conductivity of 1019 S/cm. This is 5 times higher than the conductivity of PEDOT: PSS fiber and a two-fold improvement over the reported values for nanometal/PEDOT: PSS based fabrics. The composite fiber has electric stability under bending test with bending speeds of 2 Hz indicating the composite fiber has good structural stability. In addition, the temperature of the composite fiber with 7 cm length can reach 76.5 °C at a voltage of 18 V. Additionally, the composite fiber shows anti-bacterial property and melting drop resistance, which pave the way for the integration of fiber-based optical and electrical sensors in the future multifunctional flexible devices.

**Supplementary Information:**

The online version contains supplementary material available at 10.1038/s41598-024-80655-0.

## Introduction

High-performance optical sensors have been widely developed to detect traces of viruses, toxins and pesticide residue on human body or food, such as fluorescent sensor^1,2^, colorimetric sensor^3,4^, plasmonic sensors^5–7^ and surface-enhanced Raman scattering (SERS) sensors^8–10^. Among them, SERS sensors have been attracted much attentions due to the unique characteristics of low detection limit, label-free and high molecular specificity^11^. Currently, there are three kinds of SERS substrates: solution^12^, rigid^13,14^ and flexible substrates^15–17^. The development of colloidal and rigid SERS substrates are limited by the complicated sample pretreatment and weak scalability. Textile fibers with flexibility, good wearability and air permeability are promising substrates for in-situ and real-time monitor which extends the range of SERS applications^18–20^.

Noble metallic nanomaterials (gold, silver, etc.) are generally employed in fiber-based sensors, which are absorbed with fibers by immersion^21^, hot press^22^, dip-coating^23,24^, screen-printing^25^, dyeing^26^, or the combination of these methods. However, the weak bonding force between nanometals and textile fibers results in the poor performance of sensors. Currently, there are two methods to enhance the interaction between fiber and nanometals, containing blend spinning^27,28^ and in-situ growth^29^. In-situ growth of nanometals on cellulose fibers can introduce relatively strong absorption as cellulose can be used as stabilizing agent^30^. Carbon fiber cloth with oxygen defects introduced by the complicated hydrophilization treatment provides growth sites for the formation of silver nanoparticles^31^. For most types of fiber, metallic nanoparticles are in-situ grown on fiber surface through weak interaction^32^. Blend spinning is a promising way which adapts various fibers for wide applications. Metallic nanoparticles doped fiber-forming spinning solution have been reported to fabricate fiber-based SERS substrates, but the sensitivity still needs to be improved^28,33^. Semiconductors have been used with nanometals to improve fiber-based sensor sensitivity while the bond between semiconductor and nanometal is a weak electric attraction^27,34^. The way of strong chemical bonding between semiconductor fibers and nanometals needs to be further explored.

Organic semiconductor polymer poly(3,4-ethylenedioxythiophene)-poly(styrene sulfonate) (PEDOT: PSS) provides possible binding sites for metallic nanoparticles which can form the strong metal-sulfur bonds. PEDOT: PSS film with good biocompatibility has been used as a metal-free SERS substrate to detect 10^− 3^ M 4-mercaptobenzoic acid (4-MBA)^35^ and Raman enhancement factor (EF) is of 2.26 × 10^3^ for methylene blue (MB) molecules^36^. However, there are few reports on nanometals/PEDOT: PSS composite fibers with high structural stability and high sensitivity for flexible SERS sensing. In addition, PEDOT: PSS shows excellent electrical properties. The electrical signals of PEDOT: PSS has been monitored to detect body temperature^37^ and strain^38^. High-conductive PEDOT: PSS has been used as an electrical conductor^38–40^. PEDOT: PSS is a promising candidate to integrate SERS and electrical sensors in future multi-functional fiber-based devices.

In this work, we fabricate Ag/PEDOT: PSS composite fiber by the wet spinning technology. The formation of Ag-S chemical bonds indicates the high structural stability of composite fiber. Under the combined effect of electromagnetic (EM) and charge transfer (CT) enhancements, the SERS detection limit of the composite fiber for Rhodamine 6G (R6G) reaches 10^–11^ M and Raman EF is of 1.3 × 10^7^. In addition, the electric conductivity, electron-heat performance, antibacterial properties and melting drop resistance of composite fibers are also investigated. The Ag/PEDOT: PSS composite fiber is anticipated to pave the way towards the development of multifunctional fiber-based sensors.

## Methods

### Materials

PEDOT: PSS particles (Zhuhai Kaiwei Optoelectronics Technology Co., Ltd) and Ag NWs (XFJ95, XFNANO) were used as raw materials for wet spinning process (SEM image of Ag NWs can be seen from Supplementary Information Figure S1). The mixture of isopropanol (IPA, Sinopharm Chemical Reagent Co., Ltd) and dimethyl sulfoxide (DMSO, Sinopharm Chemical Reagent Co., Ltd) were used as coagulation bath. Rhodamine 6G (R6G) and ethanol were bought from Sinopharm Chemical Co., Ltd (Shaanxi, China). All chemicals and reagents used in this study were analytical grade and without further purification.

### Characterization

The morphology, XPS spectra and Raman spectra of the fiber were characterized by the SEM (TESCAN MIRA LMS, Czech), X-ray photoelectron spectrometer (KRATOS, Axis UltraDLD) and Raman spectrometer (hr800, France HORIBA JobinYvon), respectively. The electrical property was investigated by obtaining the current–voltage (I–V) curve of the fiber using electrochemical workstation (CHI660e, China). The I-V curves of Ag/PEDOT: PSS composite fibers were measured at the length of 6, 9 and 12 cm respectively. The diameter of fiber can be obtained from SEM image, which is used to calculate the fiber conductivity through the following formula:

$$\sigma = \frac{1}{\rho } = \frac{I}{U} \times \frac{L}{S}$$

*σ* is the conductivity of fiber/(S/cm). *ρ* is the resistivity of fiber/(Ω · cm); *I* is the current of fiber/A. *U* is the voltage / V. *S* is the cross-sectional area of fiber/cm^2^. *L* is the length of fiber/cm.

The bending fatigue resistance of conductive fibers was tested in the three-point bending mode of fatigue test machine (FlexTest-TM-L, Hunan NanoUp Electronics Technology Co., LTD, China) by the electrochemical workstation, and the resistance change was detected in real time during the pressure test. The test was set to displacement loading with bending speeds of 2 Hz and bending degrees of 0 (initial state) and 125 (bend state). To verify the electric-heating performance, a voltage between 0 and 18 V was applied using the DC Power Supply (MP10010D, China). Thermal images were recorded by a thermal imaging cameras (Fluke PTi120, FLUKE, USA) with an infrared (IR) resolution of 120 × 90 (10,800 pixels). The antibacterial performance of composite fiber is test using the standard test method (GB/T 20944.1–2007, China). The Raman enhancement factors (EFs) of fiber-based SERS substrates were calculated by the formula of EF (EF = (I_*SERS*_ · C_*Normal*_) / (I_*Normal*_ · C_*SERS*_), where I_*SERS*_ and I_*Normal*_ are the Raman intensities of SERS and without SERS, C_*SERS*_ and C_*Normal*_ are molecular concentration to be measured during SERS detection and when there is no SERS)^41^. The laser wavelength of 532 nm, exposure time of 10 s and laser power of 0.5 mW were setted in Raman measurement.

### Preparation of Ag/PEDOT: PSS composite fibers

**Fig. 1:**
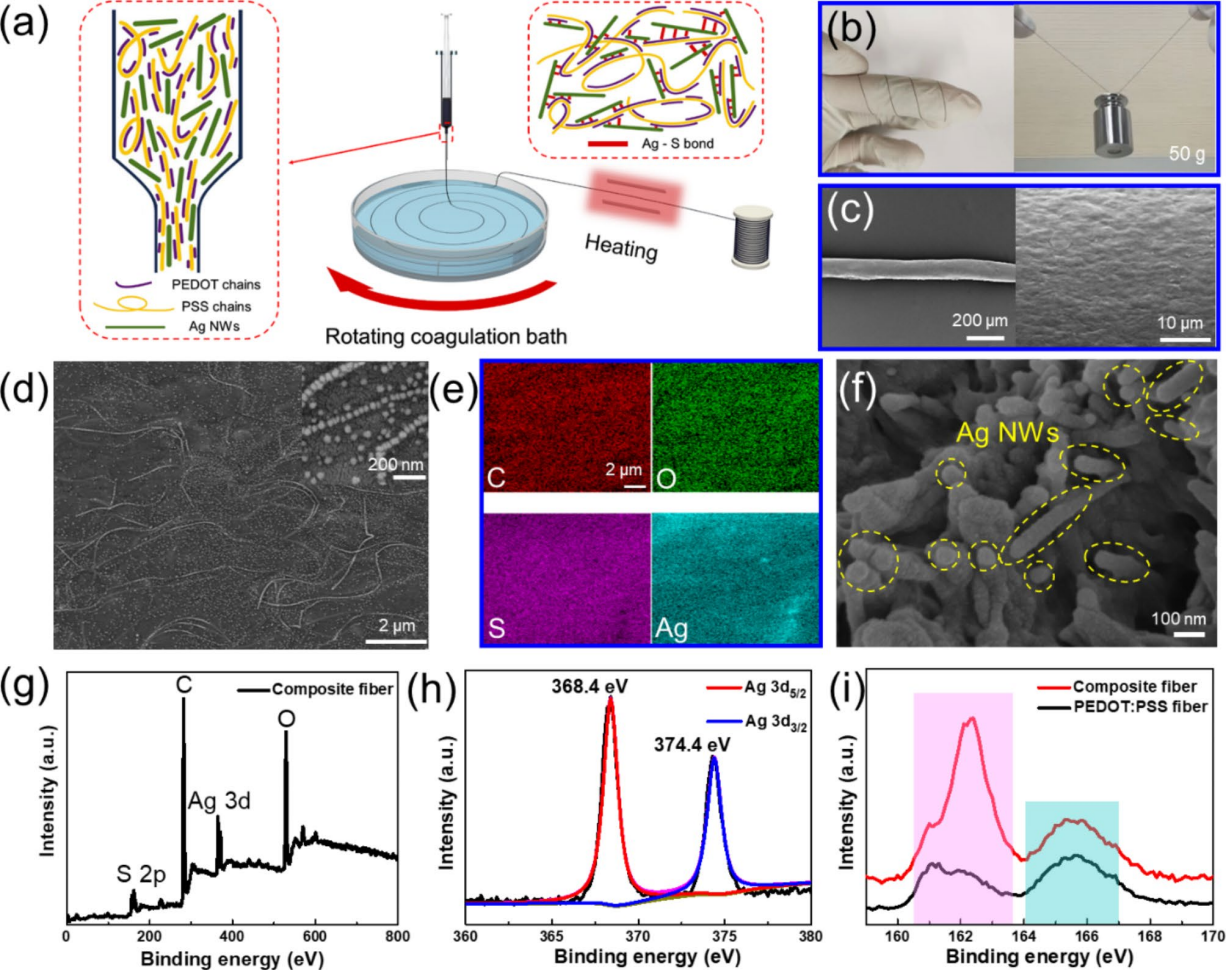
Preparation and characterization of Ag/PEDOT: PSS composite fiber. (**a**) Schematic diagram of preparation of wet-spun composite fiber. (**b**) Optical images of composite fiber on a finger and carrying a weight of 50 g. (**c**) SEM images of composite fiber. (**d**) Magnified SEM images of composite fiber. The inset shows Ag nanoparticles on composite fibers. (**e**) EDS spectra of composite fiber. (**f**) SEM image of the cross-section of composite fiber. (**g**) XPS spectrum of composite fiber. The corresponding high-resolution Ag 3d (**h**) and S 2p (**i**) XPS spectra.

Ag/PEDOT: PSS composite fibers were fabricated by the wet spinning method as shown in Fig. 1a. For more details, aqueous PEDOT: PSS dispersion (0.0526 g/ml) and Ag NWs solution with concentration of 0.0667 g/ml were prepared and stirred at 500 r/min for 30 min respectively. Then 0.6 ml of Ag NWs solution was added into 1.4 ml of PEDOT: PSS dispersion and the mixture was used as the spinning solution. The mixture of IPA and DMSO (volume ratio of 3:1) was used as coagulation bath. The spinning solution was extruded into the coagulation bath at the speed of 5 ml/h. After 24 h immersion at room temperature, the composite fiber was dried at 60 °C for 30 min for further use. The reproducibility of the preparation method can be seen from the Supplementary Information Figure S2. The produced Ag/PEDOT: PSS composite fiber can be spirally twined on a finger, and carry a weight of 50 g without fracture (Fig. 1b).

## Results and discussion

### Morphology of Ag/PEDOT: PSS composite fiber

The schematic diagram of preparation of wet-spun Ag/PEDOT: PSS composite fibers is displayed in Fig. 1a. From the SEM images in Fig. 1c, we can see the surface of Ag/PEDOT: PSS composite fiber with uniform diameter (~ 103 μm) has small grooves due to the fast diffusion between coagulation bath and solvent in spinning solution. The magnified SEM image of composite fiber in Fig. 1d shows Ag NWs are well dispersed and partially orientated in PEDOT: PSS, which can attribute to strong shear forces generated by the gradually narrowed spinning channel in the wet‑spinning process. It is found that many nanoparticles (NPs) appear at the surface of composite fiber, especially some NPs are oriented along the length direction of Ag NWs. The corresponding EDS spectra of composite fiber in Fig. 1e displays the existence of oxygen (O), carbon (C), sulphur (S) and silver (Ag) elements, indicating the composite fiber surface are decorated with Ag NPs. From the SEM image of cross section of composite fiber (Fig. 1f), we can see Ag NWs completely exist inside the fiber. Through tracing the fabrication process of composite fiber, it is found that Ag NPs are formed on composite fiber surface after the drying process (see Supplementary Information Figure S3). We infer that the fiber structure with Ag NWs inside and Ag NPs outside results from the interaction of Ag and PEDOT and the strong force applied to Ag NWs on fiber surface during drying. To further examine the attachment of Ag to PEDOT: PSS, XPS spectrum of composite fiber is displayed at Fig. 1g showing the different peaks of O, C, S and Ag elements. The high-resolution Ag 3d XPS spectrum is presented in Fig. 1h. Two peaks positioned at 368.4 eV and 374.4 eV are attributed to the binding energies of Ag 3d_5/2_ and Ag 3d_3/2_. The high-resolution S 2p XPS spectra of composite fiber and pure PEDOT: PSS fiber is shown in Fig. 1i, where two pairs of sub-peaks appear with S from PEDOT at low binding energy and PSS at high binding energy respectively^42^. Notably, after doping Ag an evident shift to higher energy is found for the S 2p peak assigned to PEDOT, while the S 2p peak position of PSS remains unchanged. The peak located at 162.4 eV results from the interaction of PEDOT and Ag forming the strong chemical bond of Ag-S^43^. The formation of such a chemical bond is able to link Ag strongly to PEDOT: PSS, resulting in a high structural-stable Ag/PEDOT: PSS composite fiber.

### SERS performance of Ag/PEDOT: PSS composite fiber

**Fig. 2:**
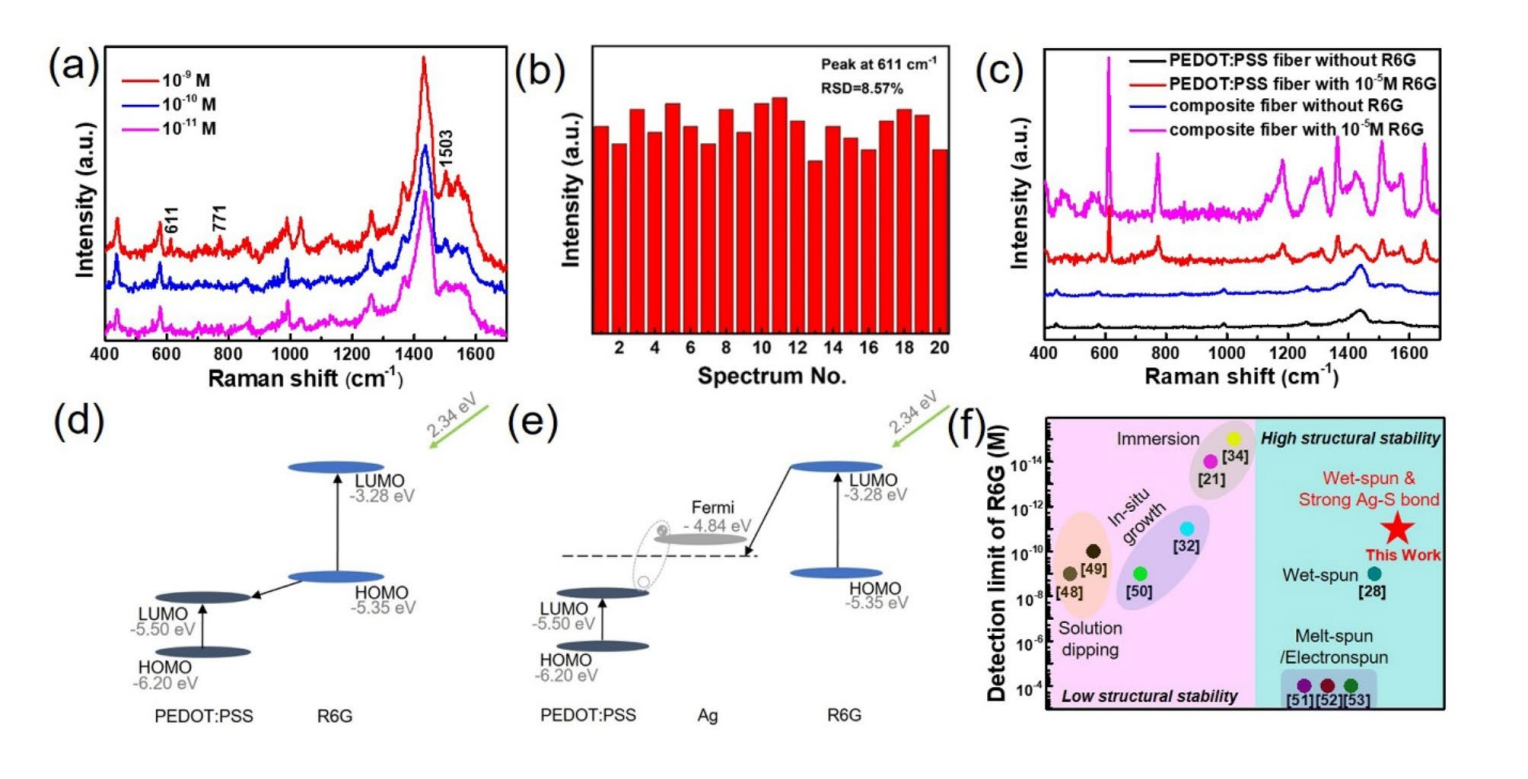
SERS performance of Ag/PEDOT: PSS composite fiber. (**a**) Raman spectra of 10^− 11^ − 10^− 9^ M R6G detected on composite fiber. (**b**) Raman spectra of 10^− 11^ M R6G on composite fiber from twenty collection points. (**c**) Raman spectra of pure PEDOT: PSS fiber and composite fiber with/without 10^− 5^ M R6G. The potential charge transfer processes in PEDOT: PSS/R6G (**d**) and PEDOT: PSS/Ag/R6G (**e**) systems under 532 nm laser excitation. (**f**) SERS detection limit of composite fiber and other reported flexible fiber-based substrate.

Ag NPs bonded with PEDOT: PSS fiber through strong chemical Ag-S bonds give possibility to high-performance fiber-based SERS sensing. Here, different concentrations of R6G molecules are selected to evaluate SERS sensitivity of Ag/PEDOT: PSS composite fiber, as shown in Fig. 2a. This selection is motivated by the strong resonant Raman effect exhibited by R6G molecules within the visible spectrum. Distinctive peaks (611 cm^–1^, 771 cm^–1^ and 1503 cm^−1^) associated with R6G are marked. The decreased Raman intensity are visible with decreasing R6G concentration, and the detection limit can reach as low as 10^− 11^ M. The spectral feature located at 611 cm^− 1^ remains unobscured by the presence of PEDOT: PSS peaks, rendering it the most optimal selection for assessing the Raman EF of the composite fiber. The calculated Raman EF is 1.3 × 10^7^, indicating an exceptionally high level of performance for the textile fiber-based SERS substrate. Figure 2b shows the intensity distribution histogram corresponding to R6G peak at 611 cm^− 1^ when R6G molecules with concentration of 10^− 11^ M are measured at 20 random points on composite fiber. The relative standard deviation (RSD) is calculated at 8.57%, which demonstrates the good homogeneity of composite fiber.

For comparison, neat PEDOT: PSS fiber is prepared by wet spinning of an aqueous PEDOT: PSS dispersion. Figure 2c displays the characteristic Raman peaks of pure PEDOT: PSS fiber and composite fiber with/without 10^− 5^ M R6G. Both pure PEDOT: PSS fiber and composite fiber with 10^− 5^ M R6G show obvious signals at 611 cm^− 1^, while the peak intensity of composite fiber is about 3 times higher than that of pure PEDOT: PSS fiber. From the finite-difference time-domain (FDTD) simulation results (see Supplementary Information Figure. S4), the SERS enhancement of composite fiber caused by the EM enhancement of Ag NPs is relatively weak, which is at least 2 orders of magnitude poorer than other dense metallic NPs with small gap^21,44^. The SERS enhancement of composite fiber can be mainly attributed to the EM enhancement of PEDOT: PSS^36^ and the photo-induced CT processes occurring in PEDOT: PSS/Ag/R6G. The possible CT in the PEDOT: PSS/R6G system and PEDOT: PSS/Ag/R6G system, induced by 532 nm laser excitation are illustrated in Fig. 2d,e. The energy levels of the highest occupied molecular orbital (HOMO) and the lowest unoccupied molecular orbital (LUMO) levels for R6G are quantified at − 5.35 eV and − 3.28 eV respectively^45^. The energy levels corresponding to the HOMO and the LUMO of PEDOT: PSS are measured at − 6.20 eV and − 5.50 eV, respectively^46^. The Fermi level of Ag is determined to be at − 4.84 eV (vs. vacuum level)^47^. For the PEDOT: PSS/R6G system under 532 nm laser excitation, the electrons located in HOMO level will absorb the energy of photon and be excited to LUMO level of R6G. Moreover, the electrons located in HOMO level of R6G can relax to the LUMO level of PEDOT: PSS. On the other hand, the electrons located in HOMO level of PEDOT: PSS will absorb the energy of photon and are excited to LUMO level.

For the PEDOT: PSS/Ag/R6G system, when Ag and PEDOT: PSS are in contact, the equilibrium surface state is constructed as the Ag-S bond formed in the contact interface of composite fiber as proved in Fig. 1i and electron migration occurs from the metal to the organic semiconductor driven by the difference in work function. The electrons can be excited from the LUMO level of R6G to the LUMO level of resonance complex formed by the combination of Ag and PEDOT: PSS. Consequently, enhancements of Raman scattering from PEDOT: PSS mainly imparts high-sensitive SERS activity to the Ag/PEDOT: PSS composite fiber, which exhibits 3 orders of magnitude better than EF of other PEDOT: PSS based SERS substrates (see Table 1). In addition, the comparison of the SERS detection limit on R6G for different metal-fiber composites is shown in Fig. 2f^21,28,32,34,48–53^. The detection limit of R6G molecules on Ag/PEDOT: PSS composite fiber substrate is comparable with other metal-fiber substrates, and the structure of Ag/PEDOT: PSS composite fiber is more stable which can be prepared by a feasible and easy fabricated way.

**Table 1:** SERS performance of PEDOT: PSS based substrates.

Substrates	Analytes	Detection limit	Enhancement factor	Reference
Ag/polypyrrole @PEDOT: PSS film	Melamine	5.42 ng/mL	1.2 × 10^4^	^54^
PEDOT: PSS film	Methylene blue	/	2.26 × 10^3^	^36^
Ag nanoparticles/polypyrrole @PEDOT: PSS film	Sodium formaldehyde sulfoxylate	100 µg/mL	693	^55^
Graphene oxide/Ag@PEDOT: PSS film	Malachite green	10^− 6^ M	/	^56^
Ag/PEDOT: PSS composite fiber	R6G	10^− 11^ M	1.3 × 10^7^	This work

### Electrical properties of Ag/PEDOT: PSS composite fiber

**Fig. 3:**
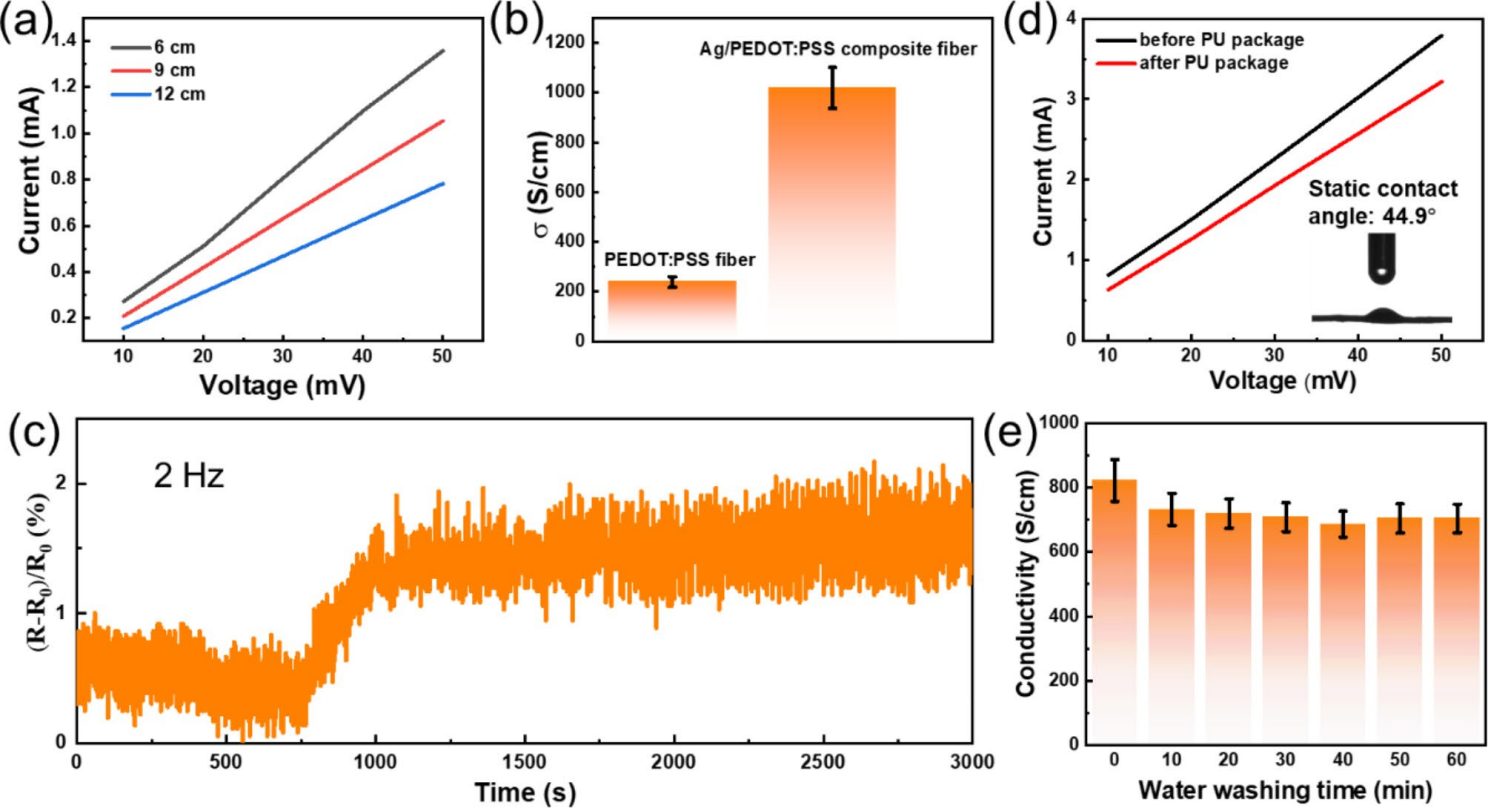
Electrical properties of Ag/PEDOT: PSS composite fiber. (**a**) I–V curves of composite fibers with different lengths. (**b**) Electrical conductivities of PEDOT: PSS fibers and composite fibers. (**c**) The electrical resistance stability of composite fiber under bending. (**d**) I–V curves of composite fibers before and after PU package. The inset shows the static contact angle of composite fiber. (**e**) Conductivity of PU encapsulated composite fiber during the water washing processes.

Both PEDOT: PSS and Ag have good electrical properties, indicating Ag/PEDOT: PSS composite fiber can be served as a conductive fiber. The I-V curves of Ag/PEDOT: PSS composite fiber with different lengths are shown in Fig. 3a. The resistance of composite fiber increases with the increased fiber length. Figure 3b displays the corresponding electrical conductivity of composite fiber, which reaches up to 1019 S/cm, 5 times higher than that of pure PEDOT: PSS fiber (~ 239 S/cm). This is, to the best of our knowledge, a two-fold improvement over the best previously reported value for nanometal-PEDOT: PSS based fabrics (426 S/cm)^57^. The bending stability of composite fiber with the highest conductivity is investigated in Fig. 3c. It can be seen that under 2 Hz bending the change of fiber resistance is increased to 0.5–1.5% at the initial stage and then kept almost unchanged even after 4000 times bending, which further indicates the composite fiber has good structural stability. In addition, the static contact angle of composite fiber is 44.9° (inset of Fig. 3d) indicating the surface of composite fiber is hydrophilic. We also check the influence of PU encapsulation on the electrical properties (Fig. 3d) and water washing performance of composite fiber (Fig. 3e). Although the fiber conductivity decreases from 1019 to 822 S/cm after PU package, the water-proof performance of encapsulated composite fiber is excellent. The electric conductivity of encapsulated composite fiber is kept at 700 S/cm after 10 min of water washing.

### Multifunctionality of Ag/PEDOT: PSS composite fiber

**Fig. 4:**
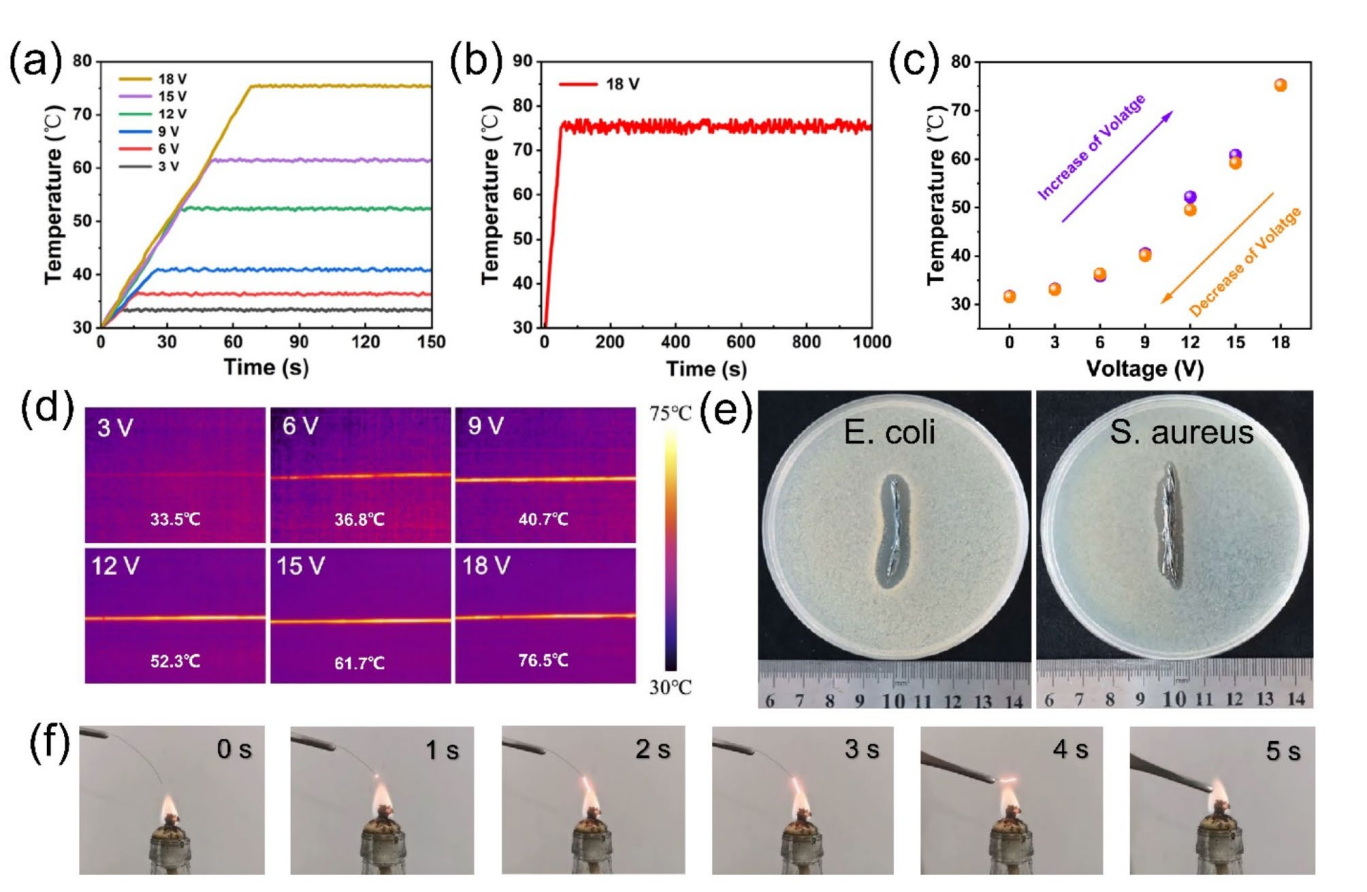
Multifunctionality of Ag/PEDOT: PSS composite fiber. (**a**) Electric heating performance of composite fiber at different driving voltages. (**b**) Temperature stability of composite fibers at a constant voltage of 18 V. (**c**) Temperature change of composite fiber under increasing and decreasing voltage. (**d**) IR images showing the saturated temperature of composite fiber with length of 7 cm at different driving voltages. (**e**) Digital images of E. coli and S. aureus cultured on composite fibers. (**f**) Digital images showing the combustion process at varying times for composite fiber.

Electrothermal performances of Ag/PEDOT: PSS composite fibers are shown in Fig. 4a–d. Figure 4a shows the electric heating property of composite fiber under varying driving voltages. The temperature of composite fiber at 3 V, 6 V, 9 V, 12 V, 15 V and 18 V can reach to 33.5 °C, 36.8 °C, 40.7 °C, 52.3 °C, 61.7 °C and 76.5 °C, respectively. As depicted in Fig. 4b, Ag/PEDOT: PSS composite fiber can be rapidly heated up to 76.5 °C in a short period of time (< 70 s) and maintain the surface temperature stable during a relative long time (> 1000 s), indicating the excellent heating reliability. At an applied voltage of 18 V, the composite fiber achieves a maximum surface temperature of approximately 76.5 °C, surpassing the threshold typically necessary for human thermal therapy, indicating the potential applications as wearable heater for human body. In addition, the composite fibers would be woven into fabrics as industrial electric heating blanket for de-icing, and surface heat sources to drive the complex, intelligent structures like shape memory alloy and polymer. When the Ag/PEDOT: PSS composite fiber is heated by increases and decreases of voltage, there is no obvious energy loss, exhibiting excellent stability and recyclability as illustrated in Fig. 4c. In Fig. 4d, the IR images further emphasize the uniformity of heat distribution within the composite fibers, which is important for electric heater. The antibacterial ability of composite fiber is explored using Escherichia coli (E. coli, gram negative) and Staphylococcus aureus (S. aureus, gram positive) as shown in Fig. 4e. Both bacteriostatic band diameters are above 1 mm indicating good antibacterial effect of composite fiber, as Ag can release silver ions to inhibit E. coli and S. aureus. In addition, the melting drop resistance of composite fiber is investigated in Fig. 4f. There is no appearance of melting drop throughout the entire combustion process. All these results indicate that the flexible, super‑tough Ag/PEDOT: PSS composite fibers with outstanding SERS performance and electrical conductivity are promising for development of textile fiber-based multifunctional flexible devices. Ag/PEDOT: PSS composite fibers can be woven into different parts of the same garment through the precise fabric design, such as the front chest for sweat SERS sensing, knee for electric heating, waist for electric conducting. Consequently, many Ag/PEDOT: PSS composite fibers can be integrated in intelligent clothing, which realizing the integration of flexible optical and electric devices.

## Conclusions

In this work, high-structural stability of Ag/PEDOT: PSS composite fibers with high-sensitive SERS sensing and high electrical conductivity are fabricated by wet spinning method via simply doping Ag NWs in the spinning solution. The interaction between Ag and PEDOT results in the formation of Ag-S bond which links Ag strongly to PEDOT: PSS. The composite fibers show low SERS detection limit (10^− 11^ M of R6G) and Raman EF (1.3 × 10^7^), which results from the synergistic reaction of EM enhancement and CT enhancement of the PEDOT: PSS/Ag/R6G system. Moreover, Ag/PEDOT: PSS composite fiber has a metal-level electrical conductivity due to the incorporation of Ag/PEDOT: PSS conductive network. The conductivity of Ag/PEDOT: PSS composite fiber is 1019 S/cm which is 5 times higher than that of pure PEDOT: PSS fiber. In addition, the saturated temperature of composite fiber can reach to 76.5 °C within 70 s under 18 V and last for above 1000 s indicating the good electrothermal performances of composite fiber. Furthermore, the good antibacterial property and melting drops resistance make Ag/PEDOT: PSS composite fiber highly promising in textile fiber-based integrated multifunctional flexible devices.

## Electronic supplementary material

Below is the link to the electronic supplementary material.

Supplementary Material 1

## Data Availability

Data is provided within the manuscript or supplementary information files.
