# Supplementary material for: Wet-spun Ag/PEDOT: PSS composite fibers for high-sensitive SERS sensing and high electrical conducting
Source: Sci Rep. 2024 Nov 25;14:29219. doi: 10.1038/s41598-024-80655-0 (PMC11589342; doi:10.1038/s41598-024-80655-0)
Supplement: Supplementary file 1 — Supplementary Material 1 [file 41598_2024_80655_MOESM1_ESM.docx]

**Wet-spun Ag/PEDOT:PSS composite fibers for high-sensitive SERS sensing and high electrical conducting**

Fan Wu^1, 2, *^, Haoyu Shi^1^, Yulong Gao^1^, Lin Cheng^3^, Tongkai Gu^4,5^, Tong Liu^1^, Ziyun Chen^1^, and Wei Fan^1, 2, *^

^1^School of Textile Science and Engineering, Xi'an Polytechnic University, Xi'an 710048, China

^2^Key Laboratory of Functional Textile Material and Product of Ministry of Education, Xi'an Polytechnic University, Xi’an, 710048, China

^3^State Key Laboratory of Dynamic Testing Technology, North University of China, Taiyuan, 030051, China

^4^School of Mechanical and Electrical Engineering, Xi'an University of Architecture and Technology, Xi’an 710055, China

^5^State Key Laboratory for Manufacturing System Engineering, Xi’an Jiaotong University, Xi’an 710054, China

^*^Corresponding author, Email: [fan.wu@xpu.edu.cn](mailto:fan.wu@xpu.edu.cn), fanwei@xpu.edu.cn

**
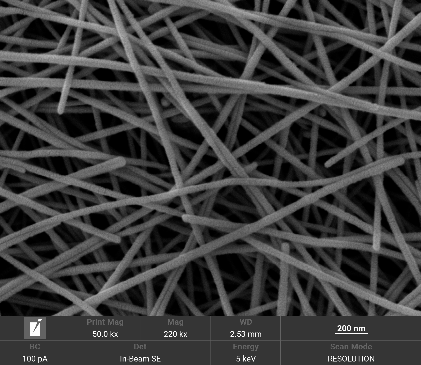
**

**Figure S1.** SEM image of Ag nanowire.

We have fabricated the Ag/PEDOT: PSS composite fibers many times using the same method described in the Experiments part. The SEM images of Ag/PEDOT: PSS composite fibers in Figure S2 indicate the reproducibility of this fabrication process.


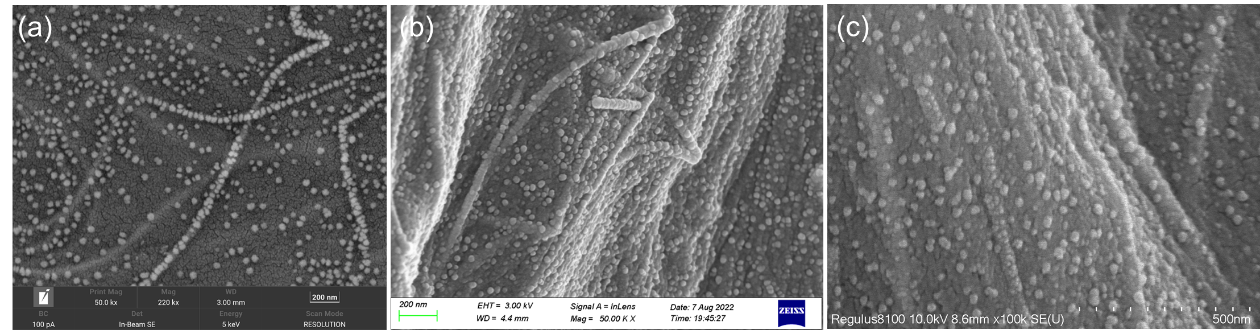


**Figure S2.** The SEM images of Ag/PEDOT: PSS composite fibers.

**
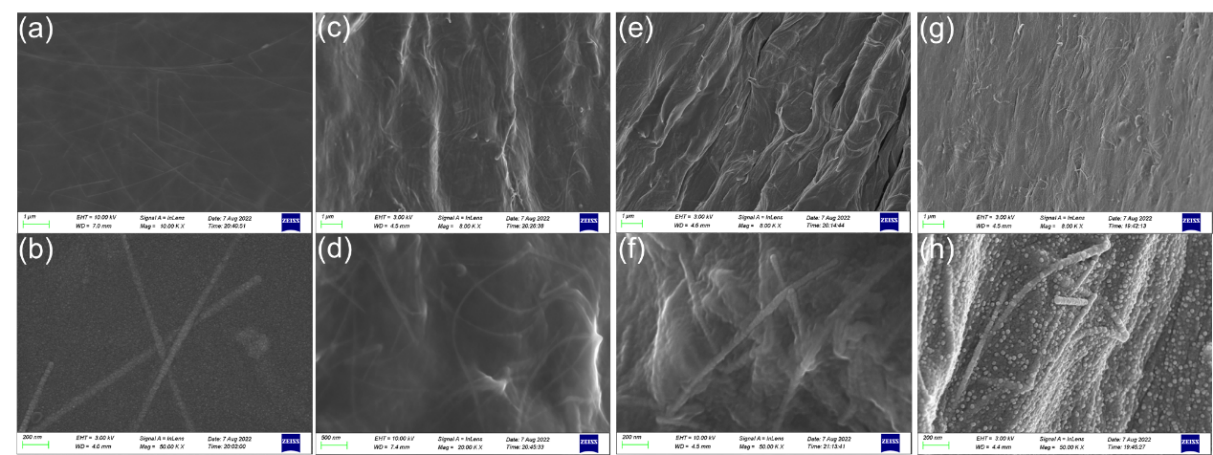
**

**Figure S3.** SEM images and magnified SEM images of Ag/ (Poly(3,4-ethylenedioxythiophene)-poly (styrene sulfonate) (PEDOT:PSS) composite fiber during the wet spinning process. (a-b) Spinning solution. Wet-spun Ag/PEDOT:PSS composite fiber with coagulating time of 5 minutes (c-d) and 24 h (e-f). (g-h) Wet-spun Ag/PEDOT:PSS composite fiber after being dried at 60℃ for 30 minutes.

To further analyze the effects of Ag NPs on SERS enhancement, finite-difference time-domain (FDTD) method is utilized to investigate the electric field distribution of Ag NPs with an excitation wavelength of 532 nm (see Figure S4). Ag NPs with the size of 38 nm and the gap between Ag NPs of 10 nm are selected in the simulation which are comparable with the experimental conditions. Electric field enhancement is low resulting from the weak coupled plasmons between Ag NPs. The maximum electric field enhancement only reaches 48 when three Ag NPs exist with the gap of 10 nm.

**
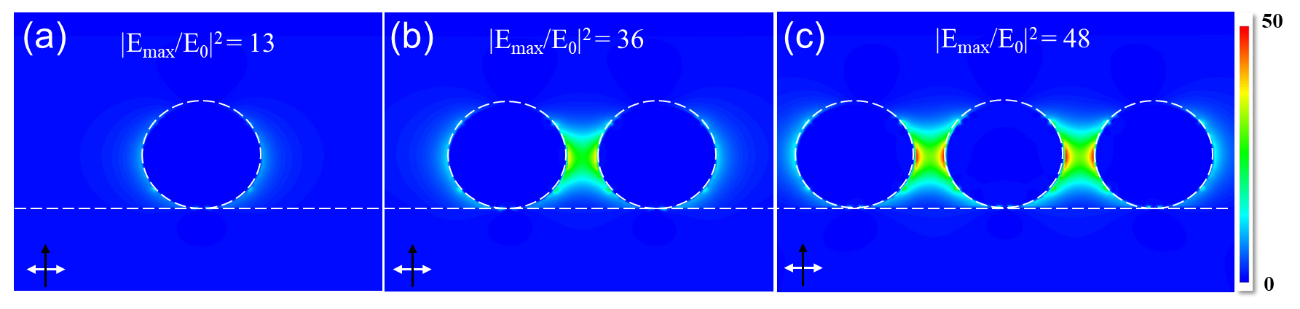
**

**Figure S4.** Electric field intensity distributions (indicated by the color bar) of nano Ag (diameter: 38 nm) on PEDOT:PSS. (a) Single Ag nanosphere. Two Ag nanospheres (b) and three Ag nanospheres (c) with gap of 10 nm respectively. The white and black arrows indicate the electric field polarization and incidence direction of excitation light, respectively.


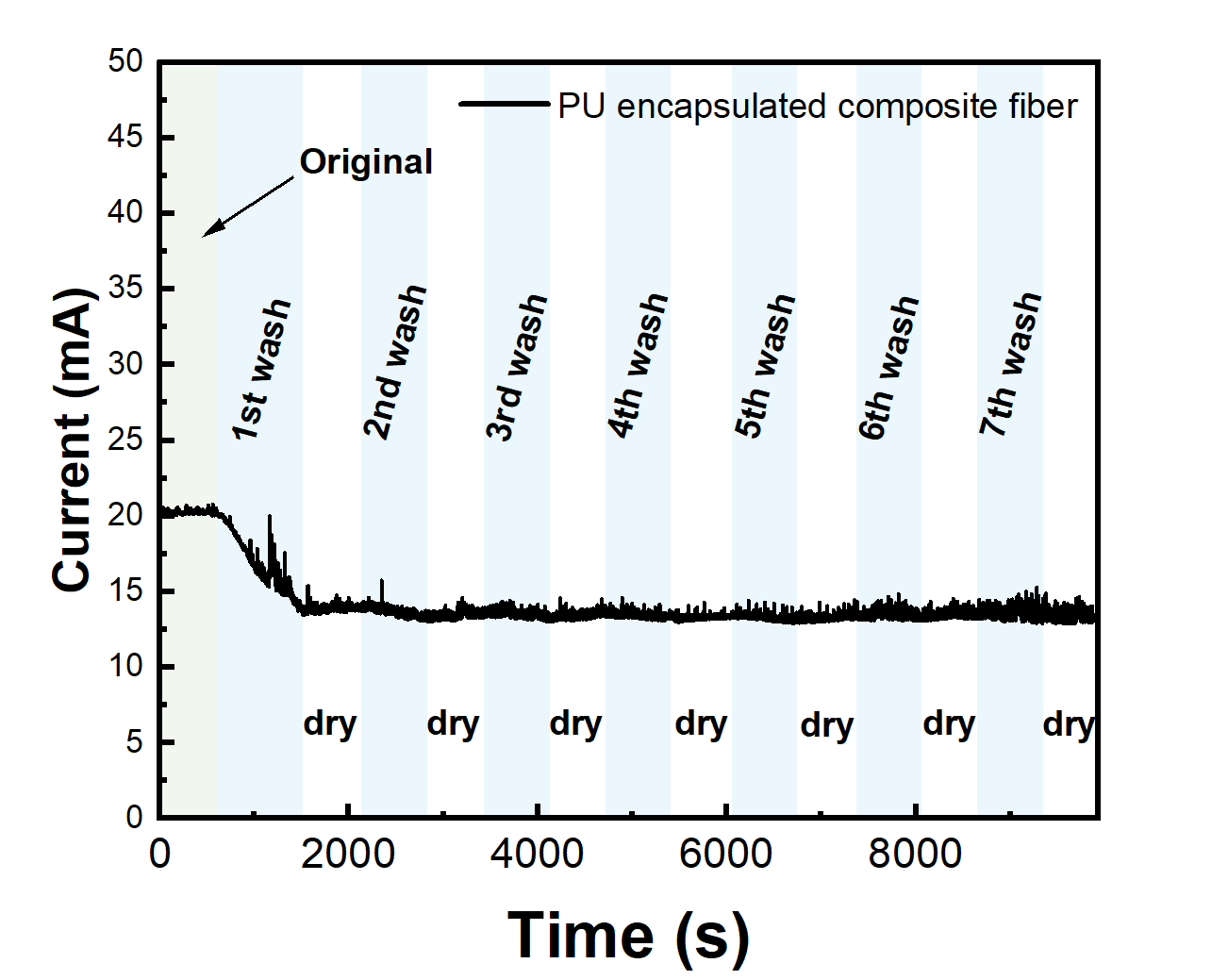


**Figure S5.** Water washing performance of Ag/PEDOT:PSS composite fiber.
